# Evaluation of GeneNAT Real-Time Polymerase Chain Reaction Analyzer and Pre-loaded Chip-Based Mycobacterium tuberculosis and Multidrug-Resistant Tuberculosis Detection in the Diagnosis of Pulmonary Tuberculosis

**DOI:** 10.7759/cureus.65067

**Published:** 2024-07-21

**Authors:** Pankaj Jorwal, Binit K Singh, Ankita Anand, Faisal Khan, Krisha Khandelwal, Neeraj Nischal, Manish Soneja, Prayas Sethi, Shikha Dhawan, Naveet Wig

**Affiliations:** 1 Medicine, All India Institute of Medical Sciences, New Delhi, IND; 2 Microbiology, New Delhi Tuberculosis Centre, New Delhi, IND; 3 Infectious Diseases, Sharda School of Basic Sciences and Research, Sharda University, Greater Noida, IND; 4 Internal Medicine, All India Institute of Medical Sciences, New Delhi, IND; 5 Public Health, SHARE (Society for Health Allied Research and Education) India, New Delhi, IND

**Keywords:** pulmonary tuberculosis, extrapulmonary tuberculosis, nontuberculous mycobacteria (ntm), eptb, poct, multi-drug resistance tb, genexpert ultra

## Abstract

Background: Tuberculosis (TB) is still the second causative agent of death worldwide after COVID-19. It is caused by *Mycobacterium tuberculosis* (MTB) infection.

Objective: The aim of the current study was to compare the performance of GeneNAT real-time polymerase chain reaction analyzer and pre-loaded chip-based MTB screening and multidrug-resistant tuberculosis (MDR-TB) detection kit (Smart Sure^TM^ MTB & MDR-TB, Genetix Biotech Asia Pvt. Ltd., New Delhi, India) against the established WHO-approved GeneXpert Ultra (MTB/rifampicin (RIF)), line probe assay (LPA), and mycobacteria growth indicator tube (MGIT) culture at point of care (POC) level.

Methods: A total of 450 pulmonary TB (PTB) suspect patients were enrolled from October 2023 to March 2024 at the Intermediate Reference Laboratory, Department of Medicine, All India Institute of Medical Sciences, New Delhi, India. GeneXpert and GeneNAT tests were done directly from sputum specimens. However, processed sputum specimens were used for both LPA (GenoType MTBDRplus) and liquid culture and drug susceptibility testing (MGIT culture and drug susceptibility testing (DST)).

Results: On comparing with GeneXpert, for the detection of MTB and rifampicin (RIF), Smart Sure^TM^ showed a sensitivity of 98.18% and 97.5% with a specificity of 100% and 98.92%, respectively. While comparing mutations in the *rpoB* gene with LPA, the Smart Sure^TM^ MDR-TB kit exhibited sensitivity and specificity of 96.77% and 99.12%, respectively. For *katG* and *inhA* genes, sensitivity and specificity were 97.6% & 85.71% and 98.66% & 98.01%, respectively, for both genes. Smart Sure^TM^ MDR-TB showed comparable results with MGIT-DST with sensitivity and specificity of 96.88% & 96.15% and 98.99% & 99.02%, respectively, for both RIF and isoniazid (INH) drugs.

Conclusion: The GeneNAT system test may provide the status of RIF and INH resistance in PTB cases in a short time with the use of minimal specimens. It required very little infrastructure with less skilled laboratory staff in comparison with other WHO-approved diagnostics used in resource-limited countries with TB and drug-resistant TB burdens.

## Introduction

Tuberculosis (TB) is caused by a gram-positive, acid-fast, rod-shaped, and aerobic bacterium called *Mycobacterium tuberculosis* (MTB) that majorly attacks the lungs but may also attack other body parts such as the kidney, brain, and spine. MTB that affects the lungs is called pulmonary tuberculosis (PTB) while rest infections are termed extrapulmonary tuberculosis (EPTB). It is one of the most ancient diseases of mankind. The World Health Organization (WHO) declared TB a "Global public health emergency" in 1993 [1]. India shares the highest burden of TB in the world with an estimated number of around 27 lakh cases in 2019 (estimated by the WHO). It is approximately 24% of TB prevalence worldwide. India also has the highest mortality due to TB, accounting for almost 21% of TB deaths worldwide. The End TB Strategy's ambitious objective of reducing TB incidence by 90% and mortality by 95% by 2035 cannot be achieved without developing new tools, such as better diagnostic tests, shorter treatment regimens, and more effective vaccines. Despite the severity of the epidemic, approximately 3 million people with TB were deemed “missing” due to underdiagnosis as well as underreporting to national TB programs [2]. A significant contributing factor to the case detection gap is the continued reliance of high-burden nations on smear microscopy and passive case detection as preliminary evaluations for diagnosis. In the world, only 33% of all the cases were initially tested by using quick molecular diagnostic assays as recommended by the WHO [3]. This is far below the End TB Strategy's target, which states that all patients notified with TB must be tested with molecular diagnostic assays as the initial test by 2025 [4].

India reported almost 27,000 multidrug-resistant tuberculosis (MDR-TB) cases, the highest in the world, and 2000 extensively drug-resistant TB (XDR-TB) cases in 2019 [5]. Under the National Tuberculosis Elimination Programme (NTEP), India has reported an increase in TB case notifications from 16% in 2019 to 22% in 2020 via the Ni-kshay portal, which ultimately aims for notification and monitoring for TB case management in India [6].

Rapidly diagnosing active PTB is crucial for both individual treatment and public health intervention to minimize further community transmission [7]. To provide timely and effective treatment, there is a requirement for rapid, affordable, and user-friendly tests to diagnose TB and drug-resistant TB (DR-TB). These tests should be deployable at the most basic healthcare levels, such as point of care (POC), and manageable by healthcare workers with minimal training. In the current scenario of clinical practice, rapid diagnosis of PTB continues to be challenging for clinicians [8].

For several decades, smear microscopy by Ziehl-Neelsen (ZN) staining has been the gold standard for the diagnosis of PTB, particularly in resource-limited and high TB-burden countries such as India [9]. Microscopy has low sensitivity [10] and is incapable of distinguishing between *Mycobacterium tuberculosis* complex (MTBC) and nontuberculous mycobacteria (NTM) strains. Following the recommendation by the WHO in 2009, fluorescent light-emitting diode (LED) microscopy, with a higher sensitivity for the detection of TB bacilli, was intended to replace ZN microscopy. However, till 2014, only 7% of microscopy centers globally were equipped with LED microscopes [11]. MTB culture in a suitable medium remains the gold standard diagnostic test. The samples can be cultured in a solid medium, i.e., Löwenstein-Jensen or liquid media Middlebrook 7H9 used in the BACTEC mycobacteria growth indicator tube (MGIT) 960 system (Becton, Dickinson and Company, Franklin Lakes, NJ). Sensitivity, specificity, contamination rates, and time to detection exhibit considerable variation across different media. The WHO recommends the dual use of systems where feasible. The significant advantage of liquid-based systems is the rapid time to detection [12]. However, inadequate laboratory facilities in resource-limited settings often limit its practical application. While culture is not recommended for use as a first-line test, it remains an important part of TB diagnostics where persistent culture positivity can predict the likelihood of relapse [13].

Considering the constraints associated with culture and direct microscopy, the WHO suggests utilizing a biomolecular test as the primary diagnostic measure for a suspected patient [14]. Currently available WHO-endorsed molecular tests are Xpert MTB/rifampicin (RIF) and Xpert MTB/RIF Ultra assays (Cepheid, Sunnyvale, CA); Truenat MTB, MTB Plus and MTBRIF Dx tests (Molbio Diagnostics, Goa, India); and GenoType MTBDRplus (Hain LifeScience GmbH, Nehren, Germany). However, these methods are accompanied by limitations of the requirement of expensive equipment with skilled labor and difficulty to perform in remote settings [15]. The WHO also endorsed GeneXpert (MTB/RIF) and line probe assay (LPA) for rapid diagnosis of rifampicin (RIF) resistant/drug-resistant (RR/DR) TB. GeneXpert provides the resistance status of RIF only and is recommended for both PTB and EPTB while LPA is recommended for only sputum specimens and provides the resistance status of both RIF and isoniazid (INH) in a single run. The current study was planned to evaluate existing diagnostics with the GeneNAT real-time polymerase chain reaction (PCR) analyzer [1] with pre-loaded chips for the detection of MTB and its drug-resistant (DR) patterns for both RIF and INH drugs in PTB cases (Smart Sure^TM^ MTB and MDR-TB kit) (Genetix Biotech Asia Pvt. Ltd., New Delhi, India]. This current system can be used at peripheral healthcare centers and resource-limited settings as a POC facility. The Smart Sure^TM^ MTB and MDR-TB kit can detect and diagnose MTB and MDR-TB and has a limit of detection (LoD) as low as two to 10 copies per sample and a sensitivity of over 96% in all laboratory-based analyses [16].

## Materials and methods

Ethical statement

The ethical approval for the study was taken from the Institute Ethical Committee, All India Institute of Medical Sciences (AIIMS), New Delhi (IEC-609/06.10.2023, RP-41/2023). All the study subjects were enrolled in the study after getting written informed consent. Study subjects who did not give consent were excluded from the study.

Study subjects

For this current study, 450 PTB-suspected patients were recruited from the medical outpatient department (MOPD) and the directly observed treatment (DOT) center of AIIMS, New Delhi, India. This was a prospective study conducted at the Intermediate Reference Laboratory (IRL), Department of Medicine, AIIMS, New Delhi, India. The National Mycobacteriology Accreditation System of Central TB Division, Ministry of Health and Family Welfare, Government of India has accredited the laboratory since 2011 for LPA (first-line and second-line antitubercular drugs) and culture & drug susceptibility test (DST). All PTB patients from October 2023 to March 2024 were enrolled in this study.

Specimen collection and processing

For this study, sputum specimens were collected at IRL in sterile containers from clinically suspected PTB patients. The collected specimens were handled at the biosafety level 3 (BSL 3) laboratory in the biosafety cabinet. Afterwards, all sputum specimens were processed for GeneXpert Ultra and Smart Sure^TM^ MTB and MDR-TB kits, irrespective of microscopy to establish the use of current molecular diagnostics at the point-of-care testing (POCT) level. The decontamination of specimens was done by N-acetyl-L-cysteine and sodium hydroxide (NALC-NaOH) method. Subsequently, the sediments were suspended in 1-1.5 ml sterile phosphate buffer (pH = 6.8). Further, all the decontaminated samples were processed for LPA as well as MGIT culture as per the protocol [17].

GeneXpert MTB/RIF

GeneXpert Ultra (MTB/RIF) was done using the Cepheid GeneXpert® system, according to the manufacturer’s instructions. At the end of the test, the result was reported as MTB detected or MTB not detected along with RIF resistance status [17].

GeneNAT TB real-time PCR analyzer procedure for MTB and MDR-TB

In GeneNAT, a minimum of 200 μl direct sputum samples were processed for the extraction of DNA using the Mini Purifier P4 extraction platform provided by the manufacturer and, subsequently, the Smart Sure^TM^ MTB screening kit or SMART™ MDR-TB detection kit was used for the detection of MTB and MDR-TB as per manufacturer instructions. This detection kit is an in vitro diagnostic (IVD) intended for the detection of MTB and MDR-TB DNA from the sputum samples by using real-time polymerase chain reaction (RT-PCR). The kit is based on a biochip format, where target-specific primers and probes are pre-labeled and employed for the detection of the MPB64 gene, IS6110 gene, *rpoB* gene, *katG *gene, and* inhA* gene specific for MTB dehydrated on chip wells to avoid contamination.

Each biochip of the Smart Sure^TM^ MTB screening kit evaluated eight patient samples simultaneously at a time along with positive control (PC) and negative control (NC). Well number 1 corresponds to the NC (contains pre-labeled primer and probe without any template), well numbers 2 to 9 include patient samples, and well number 10 is assigned to PC (contains pre-labeled primer and probe with positive template DNA) to ensure the proper functioning of premix and the chip in both the kits. In the Smart Sure^TM^ MDR-TB detection kit, one sample with NC and PC in a single run was evaluated.

In the Smart Sure^TM^ MTB screening kit, a reaction mixture of 5 μl of master mix and 5 μl of extracted DNA sample was used. For NC and PC, 5 μl of nuclease-free water was used. In the SMART™ MDR-TB detection kit, a sample reaction mixture containing 30 μl of master mix and 30 μl of extracted DNA sample was used. The prepared reaction mixture was inoculated on the chip for the detection of MTB and MDR-TB. In the MDR-TB detection kit, the biochip simultaneously detects the MTBC along with DST for both RIF and INH.

Both the available kits were designed for use on GeneNAT™ 300/GeneNAT™ 340, a real-time PCR system based on the TaqMan® probe assay. During the PCR reaction, when target-specific primers and probes are bound to the target DNA, the DNA polymerase cleaves the probe at the 5'-end and separates the reporter dye from the quencher dye of the probe through exonuclease activity. This cleavage results in the fluorescent signal generated by the cleaved reporter dye, which was monitored in real time by the PCR detection system, and the amplification curves were displayed on the screen. The standard turnaround time for the assay was 40 minutes. The result was displayed on the screen as detected and not detected along with DST results. The PC should be positive, and the NC should be negative to confirm the test validity, if not, the test is reported as invalid.

The fluorescence signal observed in the PCR detection system is directly proportional to the amount of fluorophore released and the quantity of DNA template present in the PCR. Amplification of target DNA is detected in the FAM fluorescence channel of GeneNAT™ 300/GeneNAT 340™, a real-time PCR system. Additionally, a second heterologous amplification system is included in the SMART™ MDR-TB detection kit to detect the potential PCR inhibition. This is detected as an internal control in the ROX fluorescence channel of GeneNAT™ 300/GeneNAT™ 340, a real-time PCR system. The amplification of the internal control does not limit the detection of the target DNA of MTBC.

MGIT 960 culture and drug susceptibility testing

A 500-μl decontaminated sputum specimen was inoculated in the BACTEC MGIT machine (Becton, Dickinson and Company) for a maximum of 42 days (six weeks) from the initial incubation date. Each MGIT (BBL) tube contains 7 ml of sterile 7H9 Middlebrook liquid media and a fluorescence material at the bottom of the tube quenched with oxygen; and 0.8 ml of PANTA antimicrobial supplement. The MGIT tubes in the BACTEC machine were flagged by green light for no growth and red light for growth of *Mycobacterium* on the front drawer of the BACTEC MGIT 960 machine. The positive flagged MGIT tube was undergone with the sterility check procedure on brain heart infusion agar plating, ZN stain, and capillary technique to rule out contamination. Simultaneously DST was done for both RIF and INH drugs as per guidelines [18].

Line probe assay (GenoType MTBDRplus assay)

Samples were tested using the GenoType MTBDRplus assay (Hain LifeScience GmbH, Nehren, Germany) according to the manufacturer’s instructions [19]. Testing consisted of three steps: DNA extraction, multiplex PCR amplification using biotinylated primers, and reverse hybridization. The three steps were conducted in three different rooms. After the test was completed, the DNA probe labeled strip was cautiously interpreted according to the manufacturer’s instructions. The run was deemed valid if the conjugate control and amplification control bands were present. A positive MTB control (TUB) band indicated the presence of members of the MTB complex in the analyzed sample.

Statistical analysis

Current data were analyzed using STATA statistical software (StataCorp LLC, College Station, TX). Sensitivity, specificity, negative predictive values (NPV), and positive predictive values (PPV) were calculated while comparing the diagnostics kits. LPA (GenoType MTBDRplus), GeneXpert MTB/RIF Ultra assays (Cepheid, Sunnyvale, CA), and MGIT culture DST were used as reference standards. For MGIT culture DST, the H37Rv strain was used as a reference standard.

## Results

**Figure 1:**
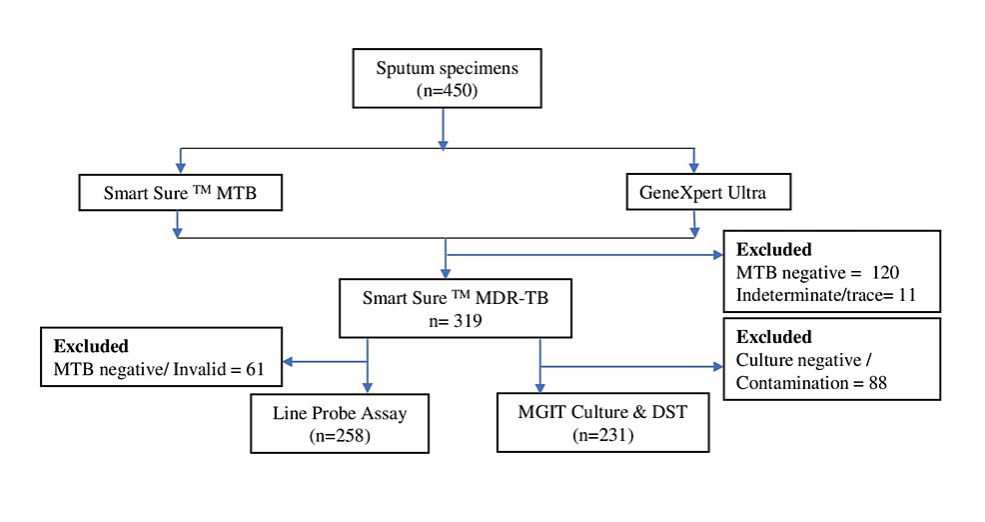
Workflow. MTB: Mycobacterium tuberculosis; MDR-TB: multidrug-resistant tuberculosis; DST: drug susceptibility testing; MGIT: mycobacteria growth indicator tube.

This study was completed in six months (October 2023 to March 2024) with a total sample size of 450 samples from the PTB-suspected patients. Among all, males and females were 206 and 244, respectively, with a mean age of 35 (±15 SD) years. All samples were sputum specimens. The primary comparison was done between the GeneXpert Ultra and Smart Sure^TM^ MTB screening kit for the identification of MTB. Both the tests identified 324 (72%) positive and 120 (26.7%) negatives for MTB. However, six (1.3%) samples were found to be negative in Smart Sure^TM^ MTB screening, which was earlier given as MTB positive by GeneXpert Ultra. Hence the sensitivity and specificity are 98.18% and 100%, respectively, as shown in Table 1. For further knowledge about RIF status, the total sample was 319 after excluding the indeterminate and trace results found in GeneXpert Ultra (Figure 1). A total of 39 samples (12.2%) were found to be RIF-sensitive by both tests. However, three (0.9%) RIF-resistant samples were shown as sensitive, and one (0.3%) RIF-sensitive was shown as resistant by Smart Sure^TM^ while comparing with GeneXpert Ultra with sensitivity and specificity of 97.50% and 98.92%, respectively (Table 2).

**Table 1:** Comparison between GeneXpert Ultra and Smart Sure MTB screening kit for the detection of MTBC (n = 450). MTB: Mycobacterium tuberculosis; MTBC: Mycobacterium tuberculosis complex; PPV: positive predictive value; NPV: negative predictive value.

MTB		GeneXpert Ultra (n = 450)			Sensitivity (95% CI)	Specificity (95% CI)	PPV (95% CI)	NPV (95% CI)
		Positive	Negative	Total				
Smart Sure^TM^ MTB	Positive	324 (72%)	0	324 (72%)	98.18% (96.08-99.33)	100.00% (96.97-100)	100.00% (98.87-100)	95.24% (90.05-97.79)
	Negative	6 (1.3%)	120 (26.7%)	126 (28%)				
	Total	330 (73.3%)	120 (26.7%)	450 (100%)				

**Table 2:** Comparison between GeneXpert Ultra and Smart Sure MDR-TB detection kit for the detection of RIF (n = 319). MDR-TB: multidrug-resistant tuberculosis; RIF: rifampicin; PPV: positive predictive value; NPV: negative predictive value.

RIF		GeneXpert Ultra (n = 319)			Sensitivity (95% CI)	Specificity (95% CI)	PPV (95% CI)	NPV (95% CI)
		Resistant	Sensitive	Total				
Smart Sure^TM^ MDR-TB	Resistant	39 (12.2%)	3 (0.9%)	42 (13.2%)	97.50% (86.84-99.94)	98.92% (96.89-99.78)	92.86% (80.82-97.57)	99.64% (97.55-99.95)
	Sensitive	1 (0.3%)	276 (86.1%)	277 (86.8%)				
	Total	40 (12.5%)	279 (87.5%)	319 (100%)				

Further, for comparison with LPA and Smart Sure^TM^ MDR-TB detection kit, a total of 258 samples were included, excluding 61 samples that were either MTB-negative or invalid in LPA. At gene-level testing, the *rpoB* gene is a surrogate marker for RIF resistance. A total of 30 (11.6%) samples were found to be resistant in both tests whereas one (0.4%) sample was found to be sensitive and two (0.8%) were marked as resistant, which were found to be discordant with LPA. Therefore, the sensitivity and specificity were 96.77% and 99.12%, respectively (Table 3). Similarly, for the INH drug, two surrogate genes *katG* and* inhA* were tagged in both LPA and Smart Sure^TM^ MDR-TB detection kit. While comparing the performance for the *katG* gene, 33 (12.8%) samples were found to be resistant by both tests whereas three (1.2%) were indicated false-positive and one (0.4%) was false-negative concerning the standard. Hence the sensitivity and specificity were 97.06% and 98.66%, respectively, as referred in Table 4. In the same way, a comparison was done for the *inhA* gene, which indicated six (2.3%) resistances by both tests while one (0.4%) specimen was additional resistant and five (2.0%) sensitive specimens were detected by Smart Sure^TM^, contradictory with the results of LPA. Therefore, the sensitivity was observed at 85.71% with a specificity of 98.01% (Table 5).

**Table 3:** Comparison between line probe assay (GenoType MTBDRplus) and Smart Sure MDR-TB detection kit for the detection of resistant patterns in the rpoB gene responsible for RIF resistance (n = 258). MDR-TB: multidrug-resistant tuberculosis; RIF: rifampicin; PPV: positive predictive value; NPV: negative predictive value; LPA: line probe assay.

RIF (*rpoB*)		LPA (n = 258)			Sensitivity (95% CI)	Specificity (95% CI)	PPV (95% CI)	NPV (95% CI)
		Resistant	Sensitive	Total				
Smart Sure^TM^ MDR-TB	Resistant	30 (11.6%)	2 (0.8%)	32 (12.4%)	96.77% (84.56-100)	99.12% (96.85-99.89)	93.75% (79.03-98.35)	99.56% (97.03-99.94)
	Sensitive	1 (0.4%)	225 (87.2%)	226 (87.6%)				
	Total	31 (12%)	227 (88%)	258 (100%)				

**Table 4:** Comparison between line probe assay (GenoType MTBDRplus) and Smart Sure MDR-TB detection kit for the detection of resistant patterns in the katG gene responsible for INH resistance (n = 258). MDR-TB: multidrug-resistant tuberculosis; INH: isoniazid; PPV: positive predictive value; NPV: negative predictive value; LPA: line probe assay.

INH (*katG*)		LPA (n = 258)			Sensitivity (95% CI)	Specificity (95% CI)	PPV (95% CI)	NPV (95% CI)
		Resistant	Sensitive	Total				
Smart Sure^TM^ MDR-TB	Resistant	33 (12.8%)	3 (1.2%)	36 (14%)	97.06% (84.67-99.93)	98.66% (96.14-99.72)	91.67% (78.11-97.13)	99.55% (96.97-99.93)
	Sensitive	1 (0.4%)	221 (85.6%)	222 (86%)				
	Total	34 (13.2%)	224 (86.8%)	258 (100%)				

**Table 5:** Comparison between line probe assay (GenoType MTBDRplus) and Smart Sure MDR-TB detection kit for the detection of resistant patterns in the inhA gene responsible for INH resistance (n = 258). MDR-TB: multidrug-resistant tuberculosis; INH: isoniazid; PPV: positive predictive value; NPV: negative predictive value; LPA: line probe assay.

INH (*inhA*)		LPA (n = 258)			Sensitivity (95% CI)	Specificity (95% CI)	PPV (95% CI)	NPV (95% CI)
		Resistant	Sensitive	Total				
Smart Sure^TM^ MDR-TB	Resistant	6 (2.3%)	5 (2.0%)	11 (4.3%)	85.71% (42.13-99.64)	98.01% (95.41-99.35)	54.55% (32.37-75.05)	99.60% (97.57-99.93)
	Sensitive	1 (0.4%)	246 (95.3%)	247 (95.7%)				
	Total	7 (2.7%)	251 (97.3%)	258 (100%)				

The mutation patterns in the Smart Sure^TM^ MDR-TB detection kit and LPA among the three target sites (*rpoB, katG*, and *inhA*) in the mentioned genes were compared. For RIF, having gene *rpoB*, two samples showing a resistant pattern in the region of *rpoB1* in the Smart Sure^TM^ MDR TB detection kit group were found sensitive (no mutation) in LPA, and rest other two groups showed concordance with LPA. In* the katG* gene, the mutation was found concordant in 36 specimens in both the Smart Sure^TM^ MDR TB detection kit and LPA whereas three specimens were found discordant (without mutation) in LPA. Subsequently, while comparing with gene *inhA*, mutations were found in 11 specimens in the Smart Sure™ kit; however, only six among those were found concordant with LPA, and the rest five were *inhA* sensitive with no mutation in LPA (Table 6).

**Table 6:** Competitive analysis of mutation patterns for Smart Sure MDR-TB detection kit with line probe assay. MDR-TB: multidrug-resistant tuberculosis; LPA: line probe assay.

Drugs	Targeted genes with mutation sites (Smart Sure^TM^ MDR-TB)		No. of samples	Sensitivity patterns in LPA	
				Resistant	Sensitive
Isoniazid	*katG* (S315T)		36 (76.6%)	33 (S315T1 = 32, S315T2 = 1)	3
	*inhA* (-8A, -8A, -15T, -16G)		11 (23.4%)	6 (C-15T, -A-16G)	5
Rifampicin	*rpoB* (L511P, D516V, S522L, H526D, H526Y, S531L)	rpoB1	2 (6.2%)	0	2
		rpoB2	0	0	0
		rpoB3	30 (93.8%)	30 (S531L)	

Additionally, the evaluation was also performed for the Smart Sure^TM^ MDR TB detection kit with MGIT culture and DST for RIF and INH drugs. A total of 231 MGIT culture-positive and MTB-positive specimens were considered for this evaluation, while 88 specimens were excluded because of either culture-negative or contamination results associated with MGIT culture. Therefore, the sensitivity and specificity for RIF were found to be 96.88% and 98.99%, respectively, and for INH, the sensitivity and specificity were observed at 96.15% and 99.02%, respectively (Tables 7, 8).

**Table 7:** Comparison between MGIT culture and Smart Sure MDR-TB detection kit for the detection of RIF resistance (n = 231). MGIT: mycobacteria growth indicator tube; MDR-TB: multidrug-resistant tuberculosis; RIF: rifampicin; PPV: positive predictive value; NPV: negative predictive value.

RIF		MGIT culture (n = 231)			Sensitivity (95% CI)	Specificity (95% CI)	PPV (95% CI)	NPV (95% CI)
		Positive	Negative	Total				
Smart Sure^TM^ MDR-TB	Positive	31 (13.4%)	2 (0.9%)	33 (14.3%)	96.88% (83.78-99.92)	98.99% (96.42-99.88)	93.94% (79.58-98.40)	99.49% (96.62-99.93)
	Negative	1 (0.4%)	197 (85.3%)	198 (85.7%)				
	Total	32 (13.8%)	199 (86.2%)	231 (100%)				

**Table 8:** Comparison between MGIT culture and Smart Sure MDR-TB detection kit for the detection of INH resistance (n = 258). MGIT: mycobacteria growth indicator tube; MDR-TB: multidrug-resistant tuberculosis; INH: isoniazid; PPV: positive predictive value; NPV: negative predictive value.

INH		MGIT culture (n = 231)			Sensitivity (95% CI)	Specificity (95% CI)	PPV (95% CI)	NPV (95% CI)
		Positive	Negative	Total				
Smart Sure^TM^ MDR-TB	Positive	25 (10.8%)	2 (0.9%)	27 (11.7%)	96.15% (80.36-99.9)	99.02% (96.52-99.88)	92.59% (75.85-98.03)	99.51% (96.74-99.93)
	Negative	1 (0.4%)	203 (87.8%)	204 (88.3%)				
	Total	26 (11.2%)	205 (88.7%)	231 (100%)				

## Discussion

The current study was performed with the objective of establishing this newer molecular diagnosis to identify the MTB as well as DR-TB at the POC level. The study evaluated the effectiveness of the Smart Sure^TM^ MTB and MDR-TB detection kit in PTB patients at a tertiary care hospital in north India at the POCT level. This was a real-time PCR analyzer with pre-loaded chip-based assays for the detection of PTB caused by MTB as well as resistance associated with RIF and INH drugs [16]. The findings of this study demonstrated promising results regarding the sensitivity, specificity, PPV, and NPV of the Smart Sure^TM^ MTB and MDR-TB detection kit (GeneNAT system), suggesting its potential as a POC tool for TB diagnosis, particularly in resource-limited settings.

The burden of TB in India remains significant, with a high prevalence of both drug-sensitive and DR-TB cases. Despite efforts to control the disease, TB continues to pose a considerable challenge to public health [20]. The study highlights the importance of improving diagnostic tools to enable timely and accurate detection of TB cases along with a very low sample volume (only 200 µl), especially in countries like India where the disease burden is substantial. However, GeneXpert, LPA, and conventional culture required a larger volume to perform the test [21].

The Smart Sure^TM^ MTB system targeted IS6110 and MPB64 genes and showed comparable results with GeneXpert Ultra, which targeted IS6110 and IS1810 genes [22] and demonstrated high sensitivity and specificity for the detection of MTB. Its low limit of detection (LoD) and ability to detect resistance to RIF and INH in a single run make it a valuable tool for TB diagnosis. The system's performance was comparable to existing molecular DST tests recommended by the WHO [23]. For RIF resistance patterns, the Smart Sure^TM^ MDR-TB detection kit also targeted the 81bp of conserved *rpoB* gene similarly with GeneXpert and LPA with the sensitivity of 97.50% and 96.77%, respectively, which is found to be acceptable with previous publications [21]. The most dominant mutation was found at position S531L (30/32; 93.8%), which had a similar association with previous publications [21,24]. Two (6.2%) mutations were not detected in LPA, showed RIF resistance, and found mutation at the rpoB1 region with the Smart Sure^TM^ MDR-TB test (Table 6).

To identify the resistance patterns in INH drug, the Smart Sure^TM^ MDR-TB detection kit also targeted *katG *and *inhA* genes, the same as LPA. Total INH resistance was found in 18.2% (47/258) of specimens. This percentage may vary from different geographical regions. While comparing mutation patterns of the *katG* gene with LPA, mutations were found in 36 (76.6%) specimens in the Smart Sure^TM^ MDR-TB detection kit, while in LPA, only 33 specimens showed mutation at S315T location in the *katG* gene, and the rest three showed sensitive. Similarly, for the *inhA* gene in the Smart Sure^TM^ MDR-TB detection kit, mutations were observed in 11 (23.4%) specimens, and only six specimens were found in concordance with LPA, and the rest five were observed sensitive in LPA (Table 6) [21,24].

In current practices in TB-prevalent countries like India, direct conventional microscopy still has been found as a backbone. It is fast and requires fewer infrastructures. However, it has low specificity toward the identification of MTB and NTM, particularly in TB-endemic countries [25]. Similarly, the WHO-approved GeneXpert and LPA required an organized setup, and instruments such as an air-conditioned lab to run the sample, especially in tropical regions of the globe where summer temperatures exceed more than 45°C [26]. At the same time, the GeneNAT system associate test required a single ventilated room to run the specific test for desired specimens. The current study underscores the need for rapid, affordable, and user-friendly tests that minimally trained healthcare workers can deploy at the POC level.

While considering the phenotypic DST (MGIT culture) as a gold-standard test, few of our specimens (88/319; 27.6%) were found to be culture negative and contaminated, and among them, 23 (23/319; 7.2%) specimens were found to be valid in Smart Sure^TM^ MDR-TB kit, limiting the utility of MGIT culture. The study's findings suggest that the GeneNAT system has the potential to address the current challenges associated with TB diagnosis, including underdiagnosis and underreporting [27]. By enabling rapid and accurate detection of TB cases, the GeneNAT system can facilitate the timely initiation of treatment, thereby reducing further transmission of the disease within the community.

The study acknowledged the limitations of the research, including the sample size and the specific setting of the study conducted at AIIMS, New Delhi, India. Further field validation and larger-scale evaluations are needed to confirm the performance of the GeneNAT system across different settings and populations. Also, this system lacks the ability to identify indeterminate or trace results (11/450; 2.4%), as observed in GeneXpert Ultra. Likewise, it is unable to detect and show the gene target site of the mutation with low sensitivity, especially in the case of the *inhA* gene, which needs to improve more. However, these advancements can promise an all-in-one test report for a better understanding of the treatment provided to the patient in the NTEP of India.

## Conclusions

In conclusion, the study found the GeneNAT real-time PCR analyzer and pre-loaded chip-based assays as a promising tool for the diagnosis of PTB and DR-TB. The system's ease of use, high sensitivity, and ability to detect resistance patterns make it a promising option for improving TB diagnostics, particularly in resource-limited settings. Further research and implementation studies are warranted to determine the system's feasibility and impact on TB control programs.
